# Toward High‐Resolution Polymeric Neural Interfaces: A Practical Guide for Electron Beam Lithography

**DOI:** 10.1002/advs.76948

**Published:** 2026-08-27

**Authors:** Hanna Karlsson‐Fernberg, Maria Asplund

**Affiliations:** ^1^ Department of Microtechnology and Nanoscience Chalmers University of Technology Gothenburg Sweden; ^2^ Science for Life Laboratory (SciLifeLab) Gothenburg Sweden

**Keywords:** e‐beam lithography, miniaturization, nanotechnology, neurotechnology, polymeric substrates

## Abstract

Electron beam lithography (EBL) offers excellent opportunities to miniaturize and refine neurotechnological implants that serve important application domains such as the treatment of neurological conditions and interface the body with external machines. To improve the biocompatibility and signal resolution of the implantable part, miniaturization of the volume and scaling up the number of channels could enable improved long‐term integration in tissue while at the same time extending the functionality. The standard is to pattern these devices using optical lithography, with a resolution limit at the microscale. In recent years, EBL has been employed to fabricate nanoscale electrical features on polymeric substrates. While plenty of data exist for EBL for standard material substrates such as silicon, challenges specific to polymeric substrates remain. These include charging of insulating materials, non‐planarity, and heating of the substrate. This tutorial supports the understanding of thin‐film micro‐ and nanofabrication process development for EBL to fabricate high‐resolution neural implants. Furthermore, it serves as a guide for implementing EBL to achieve high‐resolution structures, offering practical protocols for sub‐micron features in polymer‐based neuroprostheses, pushing this field closer to clinical implementation of these devices.

## Graphical abstract

Electron beam lithography (EBL) enables sub‐micron patterning of polymer‐based neural implants, overcoming the resolution limits of optical lithography. By addressing challenges such as charging, non‐planarity, and substrate heating, it supports higher channel density, improved miniaturization, and better long‐term tissue integration. This tutorial outlines practical fabrication strategies for high‐resolution neuroprosthetic devices and guides the broader implementation of EBL for polymeric substrates.

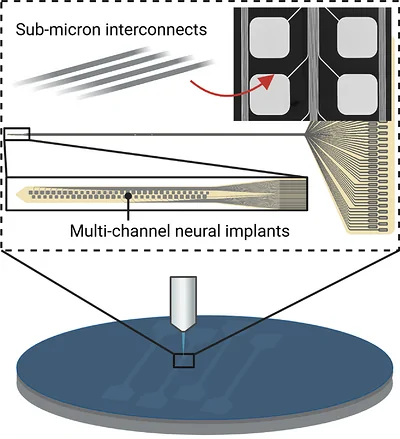

## 1 Introduction

Over the last few decades, neural interfaces have provided treatment for neurological conditions such as epilepsy and Parkinson's, and are now offering hope for the restoration of lost motor function after spinal cord injury or the replacement of lost limbs after, e.g., a traumatic injury [1, 2, 3]. By placing these devices in the vicinity of the neural tissue, they can record or stimulate electrical activity, and with the recent advances, these devices now provide bidirectional interaction between the nervous system and external hardware, facilitating, e.g., direct control of robotic prostheses (brain‐machine interface) and restoring communication for paralyzed patients (brain‐computer interface) [4, 5]. Modern, thin‐film bioelectronic devices are based on polymer films such as polyimide (PI) or Parylene C (PaC), noble metals, such as Au or Pt as interconnects, and electrode materials such as iridium oxide (IrOx) or graphene [6, 7]. A bioelectronic implant typically consists of an implanted section with electrodes able to interface directly with the neural tissue, cables to connect to external hardware, or else, a wireless interface. In addition, the interface needs a power source such as an implanted battery and external charger or power transmitter to supply energy to the battery, hardware for recording, such as low‐noise amplifiers, filters, and digital signal processing to analyze the signals [8, 9, 10]. A bi‐directional interface furthermore needs a stimulation hardware to generate constant‐current or constant‐voltage pulse generators [8]. Thus, these devices are highly complex, requiring an interdisciplinary approach to realize this technology for clinical application.

Over time, the field of implantable neurotechnology has shifted from rigid, often silicon‐based materials to flexible, polymeric materials such as PI, PaC, SU‐8, and polydimethylsiloxane (PDMS) [3, 11, 12, 13, 14, 15]. Apart from improving the mechanical matching between the tissue and the implanted device, these materials also provide electrical insulation while being chemically inert and stable, making them excellent materials for implantable neuroprosthetic devices [1, 15, 16]. Alongside material selection, further improvements in device biocompatibility have been achieved through miniaturization of the implant footprint [16]. Reduction of the overall implant footprint, together with the scaling down of critical features such as active sites and interconnects, has enabled a substantial increase in channel count, thereby enhancing signal resolution [13, 17]. This approach is particularly valuable when decoding neural activity or optimizing brain‐computer or brain‐machine interfaces, for which high spatial and temporal resolution is essential.

Contemporary implants are fabricated layer by layer using thin films, a standard method in micro‐ and nanofabrication. Lithography is a technique to transfer precise patterns onto substrates to define structures at micrometer and nanometer scales [18]. Optical methods are limited in their achievable resolution by light diffraction, which depends strongly on wavelength [19]. While optical lithography in the form of extreme UV lithography is used to fabricate nanoscale components on a mass scale, E‐beam lithography is commonly utilized in research settings for prototyping to achieve nanosized features. EBL is a flexible patterning technique as it can be used for a wide variety of materials, but it has traditionally been employed on standard semiconductor materials such as silicon, and insulating substrates, including quartz and sapphire, for the fabrication of micro components [20, 21]. In contrast, the types of polymeric substrates used in neurotechnology are less frequently patterned using EBL, with only a few examples reported in the literature, where EBL has been used mainly to achieve high integration‐density of connection lines reaching sub‐micron dimensions [12, 13].

The use of EBL on polymers poses unique challenges, including substrate non‐planarity, sample heating during electron exposure, and charging effects [12]. Importantly, process parameters can vary significantly depending on the specific polymer's properties, e.g., thermal resistance, for which more sensitive polymers, such as PaC, require more careful power control. Furthermore, as there is little data already on EBL on polymeric substrates, the process is highly dependent on the polymer, its thickness, and planarity; thus, the implementation of the process is highly individual for each case, which makes it critical to establish a stable process with a wide process window to fabricate neural implants with sub‐micron features.

Sub‐micron fabrication using EBL is an important route for increased achievable resolution on bioelectronic implants, a route not yet been extensively explored, and with only a few reports that provide detailed fabrication schemes. With this in mind, this article provides a guide to improving understanding of EBL and its general challenges, with particular emphasis on its implementation for polymeric substrates. It discusses key aspects of EBL, important resist properties, process parameter optimization, and the achievement of high‐resolution features on materials relevant to bioelectronics and neurotechnology. More general fabrication approaches for polymer‐based thin‐film neural implants have been well established and described in the literature [11, 22].

## 2 E‐Beam Lithography

**FIGURE 1:**
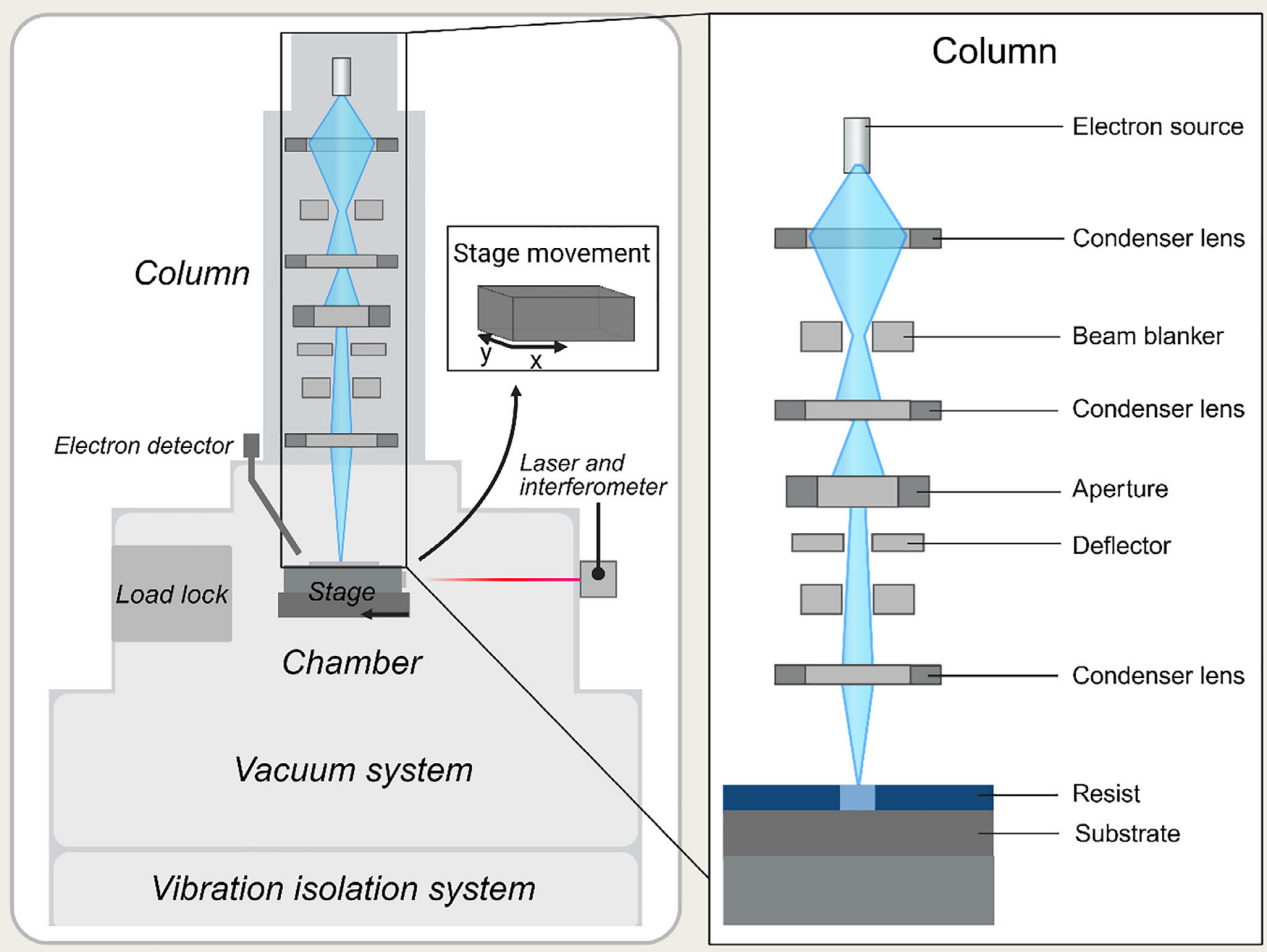
(a) A schematic of the EBL system consisting of a column, chamber that consists of a load lock, electron detector, and stage, a vacuum system, and a vibration isolation system. (b) The column consists of the electron source, several condenser lenses, a beam blanker, deflector, and an aperture.

The EBL system features a column containing the e‐beam source, a chamber for inserting and exposing the samples, a load lock, and vacuum systems to provide high vacuum (shown in Figure 1a,b) [23]. The EBL system is placed on a vibration‐isolation system to ensure the precision required for nanoscale patterning. The column consists of an electron source, either thermionic or field emission, that generates electrons accelerated to the writing voltage [19, 24]. Two or more condenser lenses whose role is to form, shape, and control the diameter of the electron beam, a beam deflector that uses an electrostatic field to precisely steer the beam, a beam blanker for turning the beam on and off, a stigmator for correcting any astigmatism in the beam, and apertures for controlling the beam and limiting the beam's divergence. The final objective lens focuses the beam onto the substrate surface. It also contains an electron detector for focusing and locating marks on the sample to align the substrate.

During exposure, the stage holds the sample and translates in the *x*–*y* plane with nanometer precision. Patterns are written in a small field, and the stage remains stationary while the beam scans each field, typically in vector mode. Because arbitrary polygons cannot be directly written, layouts are fractured into simpler shapes, e.g., rectangles and trapezoids. The division into write fields reflects the limited deflection range of the beam, with the adjacent fields later stitched together, with stage movements between fields controlled by interferometric or laser positioning [23].

EBL is inherently slow due to its serial exposure. Writing speed depends primarily on beam current, which ranges from picoamperes to nanoamperes. Higher currents increase throughput but exacerbate proximity effects (described in Section 2.2.1 Proximity effect), while lower currents improve resolution at the expense of speed. The dwell time per pixel scales with the dose, defined as the charge per unit area (µC/cm^2^), which depends on resist sensitivity, thickness, and substrate properties (e.g., backscattering from dense substrates) [19]. Pattern density also influences dose, as increased scattering in dense features reduces the required exposure. The beam current also influences beam spot size, where larger currents produce larger spots [18, 19, 23]. The beam time can be calculated from the dose, exposed area (*A*), and beam current (*i*) according to Equation (1) [18].

(1) $$t=\frac{dose\cdot A}{i}$$

Practically, this means the manufacturing times using EBL are incomparable to those achieved with, e.g., photolithography, a limitation often brought up as an argument against using EBL beyond small‐scale prototyping. For instance, one neuroelectronic device, with a total exposed area of approximately 0.42 cm^2^(including interconnects, electrodes, and pads), requires an exposure time of about 3.5 h when using a dose of 300 µC/cm^2^and a beam current of 10 nA. For a full 4‐inch wafer, the total exposure time can therefore extend 1 or 2 days, highlighting the limited throughput of EBL for this application.

In addition to throughput constraints, prolonged exposure times can introduce thermal effects that might lead to beam drift (described in Section 2.2.3 Beam drift), further compromising pattern fidelity. Strategies to mitigate exposure time, including optimization of the beam spot size, are presented in Section 4.2.3 Optimizing beam spot size.

### 2.1 Achieving High Resolution With EBL

When the electron beam interacts with the resist, electrons undergo elastic and inelastic scattering [18, 19]. Elastic scattering deflects electrons via atomic nuclei, producing forward and backscattering, while inelastic scattering transfers energy to the resist, generating low‐energy secondary electrons that primarily drive exposure within a few nanometers of the beam path. In contrast, forward‐scattered electrons spread over tens of nanometers, and backscattered electrons can travel several micrometers from the point of incidence [19, 23].

**FIGURE 2:**
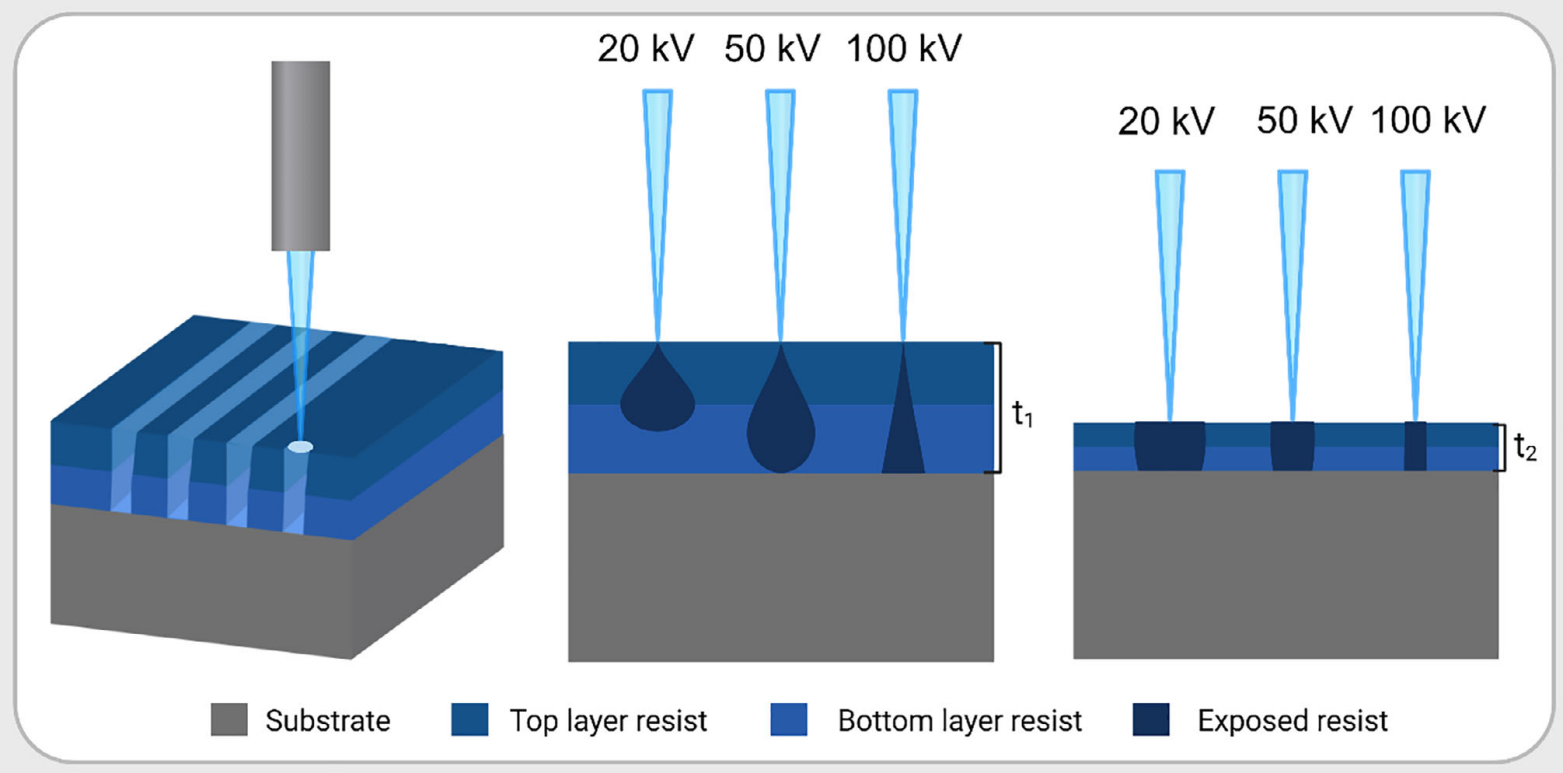
The electron interaction volume at different acceleration potentials, and the effect of using a thinner resist at different acceleration potentials. The exposure causes unintended exposure, referred to as the proximity effect. With a higher acceleration potential, the interaction volume is smaller, especially when using a thinner resist, facilitating the fabrication of high‐resolution structures.

The acceleration voltage determines electron energy and interaction volume [18, 19]. Higher voltages increase penetration and reduce forward scattering but enhance backscattering, whereas lower voltages confine exposure near the surface, improving resolution but reducing exposure uniformity. Figure 2 illustrates how varying the acceleration voltage affects the electron–resist interaction, as well as how the interaction volume changes with thinner resists.

The resolution in EBL is governed by beam diameter (spot size), step size, aperture, and acceleration voltage [19, 25]. The beam diameter depends on electron optics and beam current: higher current increases spot size, while higher accelerating voltage improves focus and reduces it. Hardware factors, including condenser lenses, apertures, and electron source type, also influence performance [18, 19, 23]. Under optimized conditions, the beam can be focused down to approximately 1–2 nm [26].

Resist properties further limit resolution. High‐contrast, low‐sensitivity resists, thin films, and optimized processing enable finer features [19], and pattern density and substrate properties also have an impact on the final resolution [27].

### 2.2 EBL Challenges

There are several challenges associated with EBL exposure, which will be briefly outlined and discussed here, namely the so‐called “proximity effect,” line edge roughness (LER), and beam‐drift.

#### 2.2.1 Proximity Effect

The scattering of electrons causes non‑uniform resist exposure and reduced pattern resolution, known as the proximity effect, a main limitation of EBL [23]. To mitigate this, proximity effect correction (PEC) compensates for scattering through exposure parameter adjustments to obtain uniform resist development [23]. Common approaches include dose correction, pattern size compensation, and background exposure compensation. In dose correction, the exposure dose is algorithmically adjusted to equalize the effective energy distribution across the pattern. Pattern size compensation provides a simpler alternative, where dense features are designed narrower and isolated features wider to offset local exposure variations, suitable for periodic patterning [23]. Background exposure compensation employs a secondary low‑dose exposure of the inverse pattern to counter backscattered electrons, eliminating algorithmic correction but reducing image contrast and increasing total exposure time.

As illustrated in Figure 2, increasing the acceleration voltage decreases the exposure volume within the resist, mitigating the proximity effect [19]. Similar improvement is achieved by reducing the beam spot size.

**FIGURE 3:**
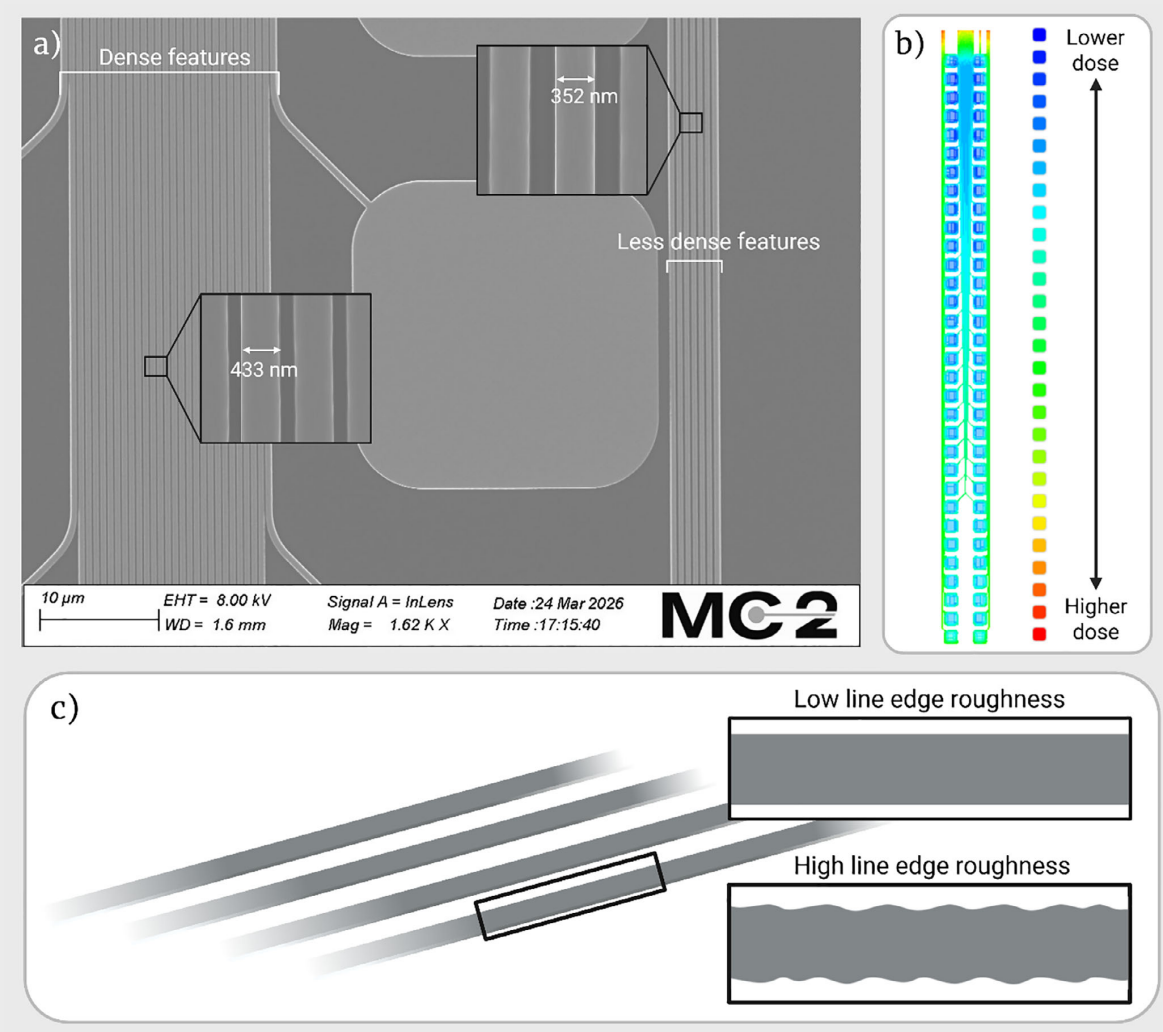
(a) A fabricated sample without proximity effect correction. The denser lines are substantially larger (∼433 nm, assessed manually with SEM) than intended (300 nm). The sparser lines are also slightly larger (∼350 nm) than intended. (b) PEC by adjusting the doses to different sections of the pattern, as well as how much the dose is adjusted represented by different colors. (c) Schematic of interconnects with low and high LER and how measurement points are taken.

Structures fabricated without any PEC with an acceleration potential of 100 kV are shown in Figure 3a. But the fabricated lines in the middle are substantially denser compared to the lines to the right. The lines are intended to be 300 nm wide but are instead approximately 430 nm wide in the denser section and 350 nm in the less dense area, showing how a denser design results in larger features due to the electron scattering. Figure 3b shows how the proximity effect can be corrected by adjusting the dose. The color indicates how much the dose is adjusted, with the darker blue representing a lower dose while the red represents a higher dose.

#### 2.2.2 Line Edge Roughness

LER refers to the deviation of the edge of the patterned feature from the ideal straight shape after exposure and development of the resist (shown in Figure 3c) [19]. For very small features (mainly 100 nm or less), this effect can have a large impact on the quality of the structures, which might significantly affect the achievable minimum feature size for a given process and maximum circuit density for devices [28]. This effect arises due to resist molecular nonuniformity, electron scattering in the resist, or fluctuations in the delivered dose (shot noise) [26, 28, 29].

To achieve high resolution with e‐beam, LER must be limited [29]. Factors affecting LER include exposure parameters, such as beam energy and dose, as well as resist and its thickness [29, 30]. The higher the beam energy and dose, the less shot noise is imparted to the delivered electrons. Thinner resist layers reduce LER by shortening the electron scattering path, which minimizes forward scattering and improves edge definition, while thicker resists increase blur and local dose variation, thereby exacerbating roughness [29].

#### 2.2.3 Beam Drift

**FIGURE 4:**
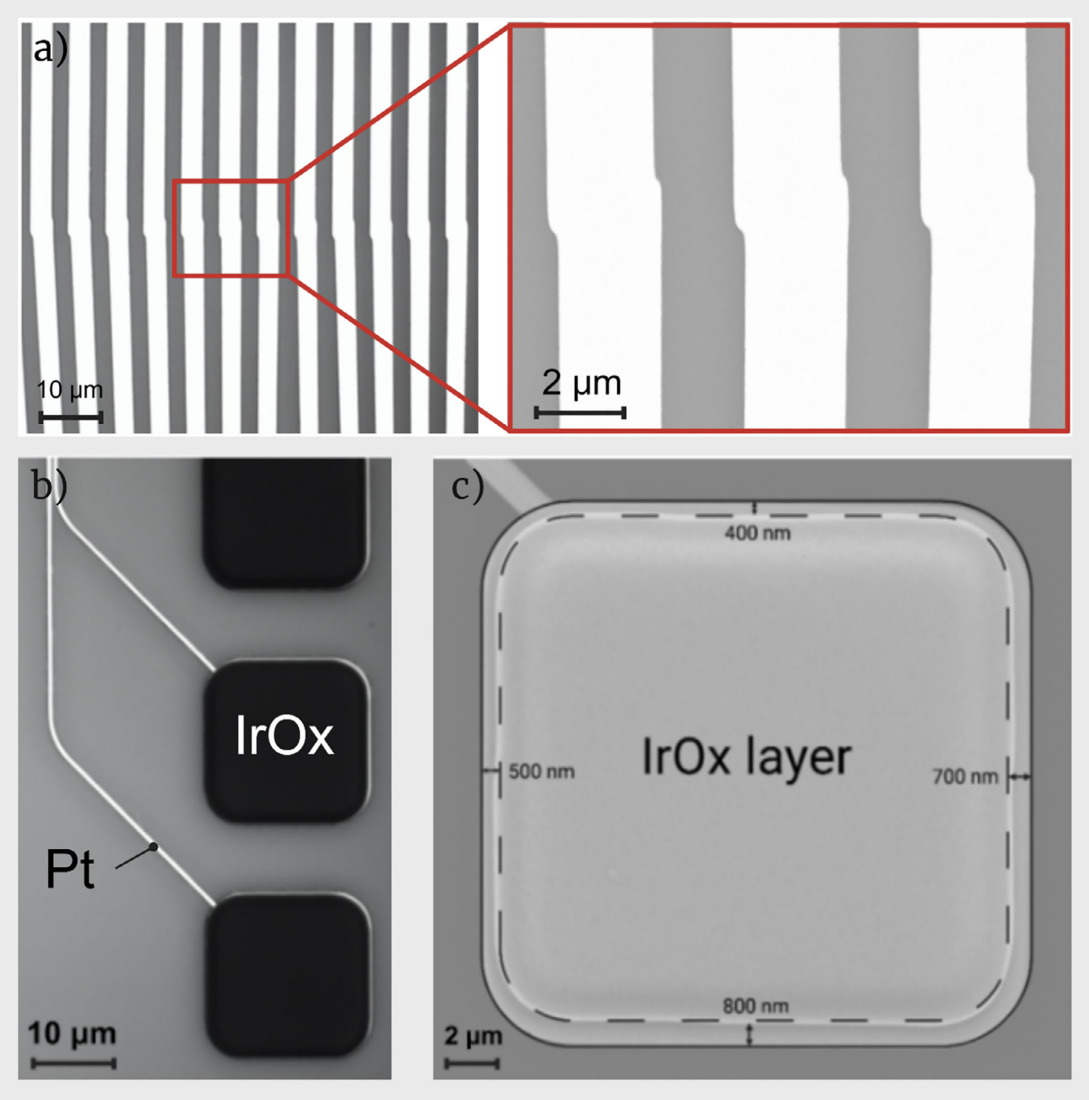
(a) Misaligned overlap due to drifting of the electron beam during exposure. (b) Optical micrograph of electrodes with a misaligned IrOx layer on top of a platinum electrodes. The misalignment possibly comes from drifting of the electron beam during (long) exposures and/or overlay errors. (c) SEM micrograph of a misaligned IrOx layer deposited on the electrode. The deposited IrOx layer is shifted to approximately 100 nm in x direction and 200 nm in y direction.

Beam drift can be induced by mechanical motions, column charging, magnetic field interference, and thermal effects [31]. Since this effect accumulates with time, the drifting is more apparent for longer exposures (>30 min), negatively affecting the pattern at the transition between writing‐fields. Beam drift can have a large impact on very small structures and substantially affect overlays in successive layers. Figure 4a shows the misaligned overlap stemming from the same exposure, likely contributed by beam drift. The design is exposed in two sequences with 1–2 h of exposure time, causing beam drift and resulting in misalignment. Figure 4b,c shows the misalignment between successive layers, showing the possible effect of beam drift. Thus, to reduce this effect across successive layers, the alignment marks should be preferentially exposed early before beam drift has had a substantial impact, and the layers should be designed with some compensatory overlap that minimizes the consequences to device functionality from such structural imperfections. Another strategy to minimize beam drift is to reduce exposure time by using a beam spot size optimized for the feature sizes (described in Section 4.2.3 Optimizing beam spot size) or using resist that can be exposed with lower doses (described in Section 4.1.1 Selection of resist and spinning parameters).

## 3 EBL on Polymeric Substrate

Multiple process pathways can be employed to fabricate thin‐film polymer bioelectronic devices [11, 22]. As this has been extensively detailed elsewhere in the literature, it is only briefly outlined here for easy reference. Metallization patterning is typically achieved through either lift‐off or etching. While both approaches are viable, lift‐off is more commonly used due to its versatility, as the same process can be applied across a wide range of metallization materials.

**FIGURE 5:**
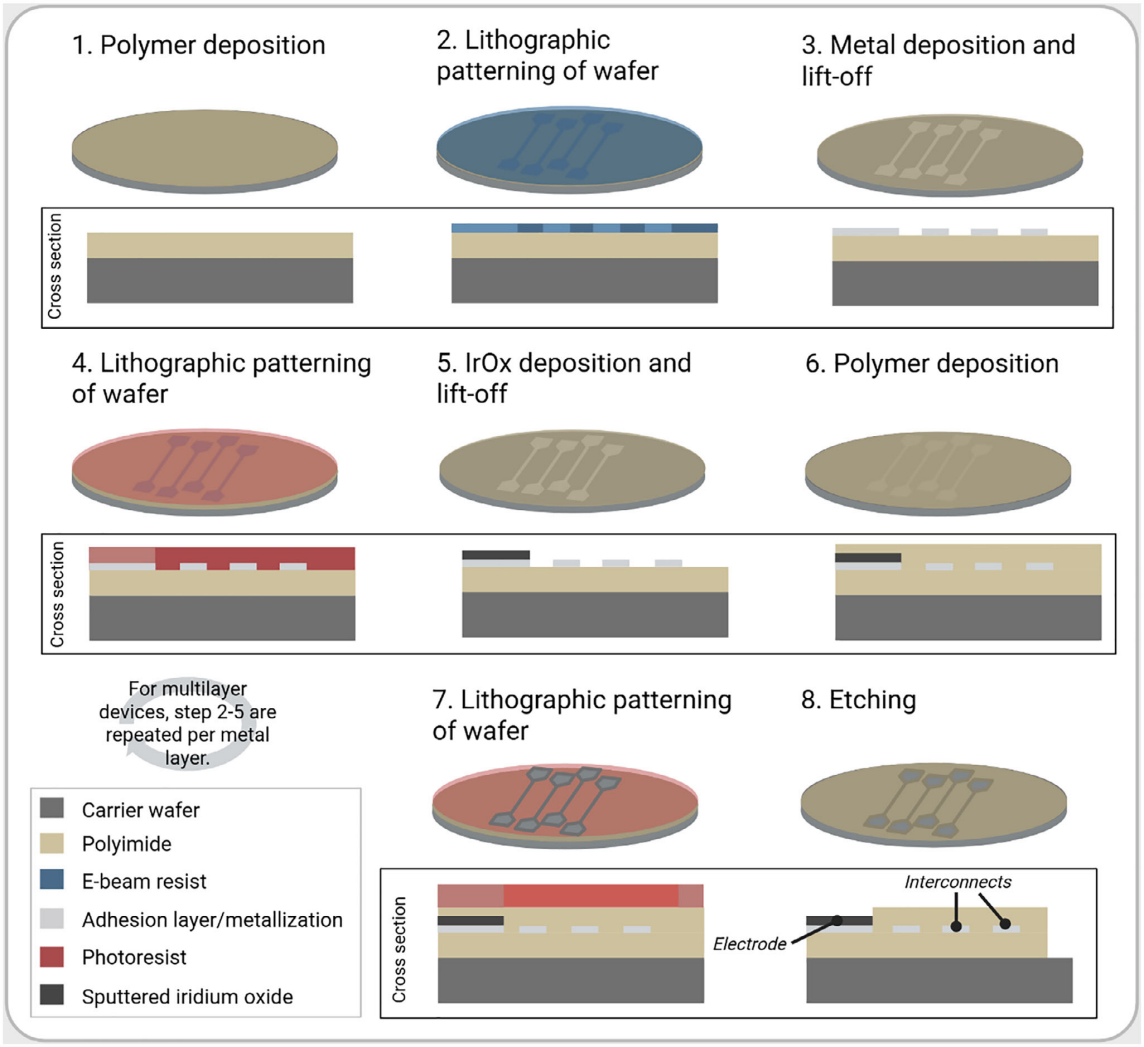
The typical fabrication process of bioelectronic devices using lift‐off starts with polymer substrate deposition, typically by spin‐coating a precursor solution followed by curing. The wafer is then patterned using lithography, after which metal deposition (adhesion layer and metallization) and lift‐off define the interconnects and electrodes. Another lithography steps define the electrodes, and IrOx is sputtered to form SIROF on the electrodes, together with additional metal stacks for improved adhesion. The metallization layer is subsequently encapsulated in another polymer layer. This process is repeated as required for multilayer structures. In the final steps, the etch layer is patterned lithographically and etched to open the device outline, pads, and electrode sites, after which the devices are released from the wafer.

A typical example of a manufacturing scheme is to, as a first step, deposit the polymer substrate on the wafer, usually by spin‐coating a precursor solution onto the wafer, followed by curing for 10 min under a nitrogen atmosphere (YES‐PB6, Yield Engineering Systems Inc.), creating a polymer layer with a thickness in the order of a few microns (Step 1, Figure 5). The metallization pattern is patterned using EBL or optical lithography, a metal and adhesion layer is deposited, and excess material is subsequently removed through lift off (Steps 2 and 3 in Figure 5). To enhance the performance of small electrodes, there are usually additional electrode layers added, e.g., a sputtered IrOx film (SIROF) (Steps 4 and 5, Figure 5).7 For devices with only one metal layer, the second layer of polymer is deposited onto the metallized structures and cured (Step 6, Figure 5), and the outlines of the wafers, electrodes, and pads are patterned using the optical lithography technique (Step 7, Figure 5), and then opened using reactive ion etching (RIE) (Step 8, Figure 5). Stacking of interconnects enables an increased electrode density per unit area by accommodating a greater number of routing lines within the same footprint. This can be achieved by depositing additional metal layers separated by polymer layers, thereby forming multilayer device architectures [11]. In this case Steps 2–5 are simply repeated before lithographic patterning and etching (Steps 7 and 8, Figure 5). When fabrication is finished, each sample can be released from the wafer, either by mechanical peeling or by dissolving a sacrificial layer beneath it.

The use of EBL on polymeric substrates is still rather unexplored, and there are numerous challenges associated with the characteristics of polymers, such as high surface‐ and volume resistivity and thermal resistance, which can cause charging and heating of the substrates. While Parylene exists in different variants, PaC is the type used mostly in biomedical applications and is thus the one that will be considered here. Likewise, with PI, here the focus is on PI2611, even though some of the tabulated values come from Kapton.

EBL exposure has been reported for PaC and PI (Kapton), as the substrate materials (marked gray in Table 1) [12, 13, 32]. SU‐8 and PDMS are both electron‐sensitive materials where EBL can be used to pattern the polymer directly, as a resist [33, 34, 35]. Thus, these substrates are less promising for patterning with EBL as the substrate. Furthermore, quartz has been patterned using EBL for decades and poses similar challenges as polymers, such as poor electrical and thermal conductivity. Thus, in Table 1, values for quartz are included for comparison. However, it should be noted that quartz is often exposed to fabricate photomasks, for which the quartz layer has a thickness in the order of a few millimeters, compared to polymeric substrates (<15 µm) on a conductive carrier wafer, which might affect the interaction of the electron beam with the substrate.

**TABLE 1:** The electrical and thermal properties of common polymers used in bioelectronic implants and quartz for comparison. The gray‐marked columns are materials that have been previously patterned with EBL as a substrate.

	Property	PDMS	Parylene C	Polyimide	SU‐8	Quartz
Electrical properties	Volume resistivity (Ω·cm)	2.9 × 10^14^[36]	8.8 × 10^16^ [37]	1.5 × 10^16^ [38]^a^	1.8 − 2.8 × 10^16^ [39]	7 × 10^7^ [40]^b^
	Surface resistivity (Ω/□)	1.78 × 10^14^ [41]	1 × 10^14^ [42]	≥ 10^16^ [38]^a^	5.1 × 10^16^ − 1.8 × 10^17^ [39]	−
	Dielectric strength (MV/m)	250–635 [43]	≈ 220 [37]^c^	∼300 [44]^d^, ^e^	440 [45]	30–37 [40]
Thermal properties	Thermal conductivity (W/m·K)	0.15–0.2 [46, 47]	≈ 0.082 [37]^f^	0.2 [44, 48]^d^	0.3 [49]	≈1.4 [40]
	Max temperature (°C)	231–400 [50, 51]	280 [52]	550 [38]^a^	300–350 [53]	>1000–1100 [54]
	Glass transition temperature (°C)	(−120)–(−125) [55]	60–100 [37, 56]	∼360–410 [56]^d^	210 21 [49]	∼1700 [40]^g^
	Linear CTE (ppm/°C)	301–310 [57, 58]	≈ 35 [37]	≈ 35 [56]^d^, ^h^	52 [49]	0.55 [40]^i^
	Specific heat capacity (RT) (kJ/kg·K)	1.46 [46]	≈ 0.7 [37]	≈ 1.09 [56]^d^	1.6 [59]	≈ 0.67 [40]

^a^ PI2611.

^b^ At 350°C.

^c^ For 25 µm.

^d^ DuPont Kapton HN.

^e^ For 25 µm.

^f^ At 25°C.

^g^ No true *T_g_*; softening at 1700°C.

^h^ From 30°C to 400°C.

^i^ 20°C–320°C.

### 3.1 Substrate Non‐Uniformity

Accurate height measurement and surface flatness control are critical to the performance and reliability of EBL processes. Deviations in sample height or local topography shift the focal plane of the electron beam, resulting in defocus, increased beam blur, and degraded critical‐dimension (CD) control, which ultimately compromises pattern fidelity and overlay accuracy at the nanoscale [60, 61]. Furthermore, the thickness and uniformity of the resist layer directly influence electron scattering and dose distribution. Thus, due to the inherent sensitivity of EBL processes as well as the variability in critical parameters of the polymer film, fabricating samples using EBL on polymeric substrates might be more complex, and thus, thorough testing is required.

**FIGURE 6:**
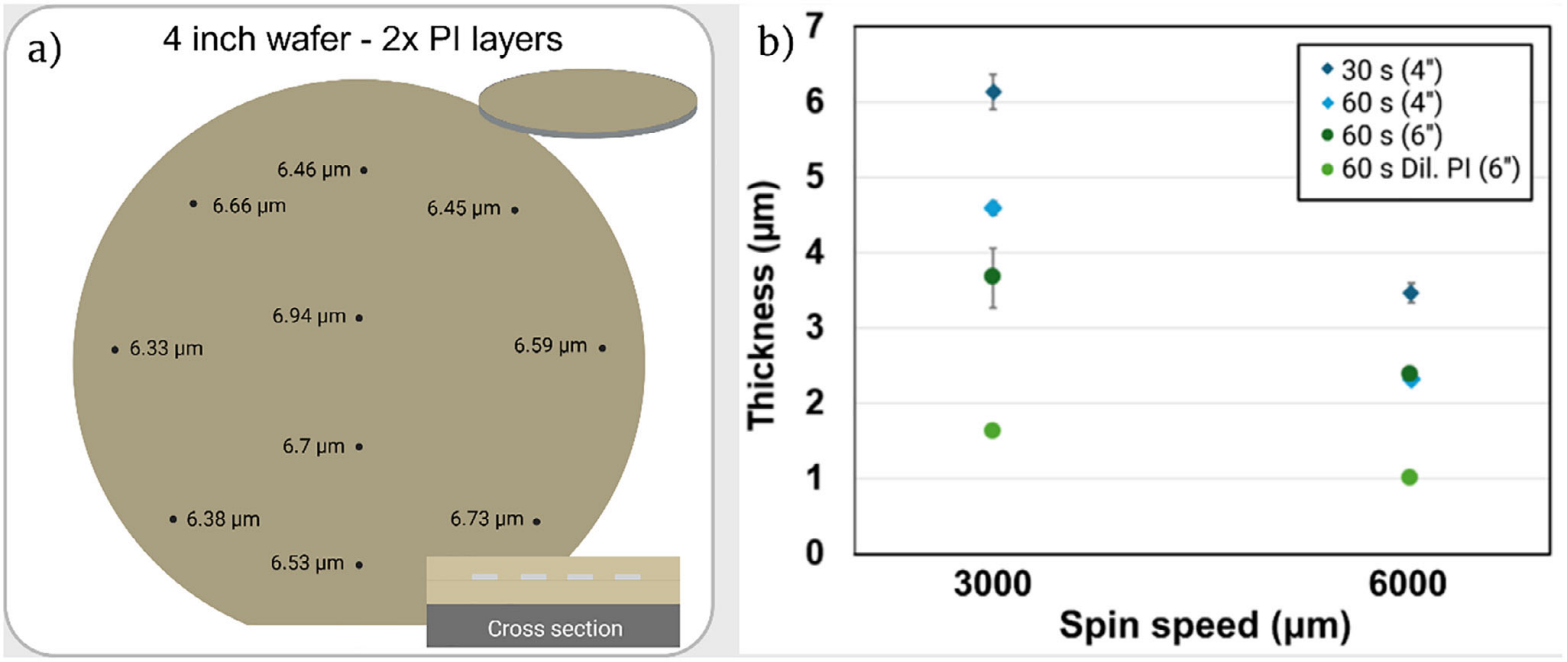
(a) Measured PI thicknesses of a 4‐inch wafer, showing the variability on the PI thickness when two layers are spin‐coated sequentially with a spin‐speed of 5000 rpm on the carrier wafer. The average thickness is 6.6 ± 0185 µm. (b) The thicknesses of one layer of PI spin‐coated at spin speeds of 3000 or 6000 rpm. When spinning PI oninch wafer for 30 s, it yields an approximately 6 µm (6.14 ± 0.47 µm, *n* = 10) thick PI layer when spinning at a spin speed of 3000 rpm and 3.47 ± 0.27 µm (*n* = 10) when spinning at 6000 rpm, while when spinning for 60 s, it yielded a 4.60 ± 0.18 µm (*n* = 10) thick layer for 3000 rpm and 2.33 ± 0.14 µm (*n* = 10) for 6000 rpm. For a 6‐inch wafer with the same spin speed, the yielded thickness was 3.67 ± 0.8 µm for 3000 rpm and 2.39 ± 0.073 µm for 6000 rpm. When diluting the PI with NMP with a ratio of 4:1, the thickness was 1.62 + −0.055 µm for a spin speed of 3000 rpm and 1.01 + −0.0562 µm for 6000 rpm.

Spin‐coating is a common deposition method of polymers used for the fabrication of neural devices. When spin‐coating, the thickness and film uniformity depend on spin‐time and viscosity of the precursor solution, as well as the size of the carrier wafer. Typical variation in film thicknesses, here shown for two PI (PI2611) layers spin‐coated with a spin‐speed of 5000 rpm on a 4‐inch wafer with a total thickness of 6.6 ± 0185 µm (shown in Figure 6a), measured using a step D‐500 Profilometer (KLA Instruments, Milpitas, CA). The data were recorded in a step‐up/down mode at a speed of 0.1 mm s−1 with a stylus force of 10.0 mg. The thickness and standard deviation of a spin‐coated layer of (spin‐speed 3000 and 6000 rpm) diluted (4:1 PI:NMP) and undiluted PI, on 4‐and 6‐inch wafers is shown in Figure 6b. The figure shows that with increased spin‐time, the precursor solution reaches a steady state, and a higher degree of film uniformity can be attained. Likewise, for smaller wafers, higher spin‐speed and diluted PI there are more polymer film uniformity. It shows that a longer spin‐time is preferred, and for larger wafer there can be more variability in the film's thickness. The variability is small even though non‐planarity in the range of a couple of hundred nanometers can be expected. Furthermore, diluting the polymer yields a thinner and more uniform polymer layer.

Acceleration is a critical parameter during spin coating, as it governs the initial spreading of the polymer across the wafer. Excessively rapid acceleration can lead to shadowing effects, resulting in non‐uniform film thickness. For highly viscous coatings such as PI, tailored spin programs are often required to ensure uniform coverage. For example, an initial low‐speed step (e.g., 500 rpm) of a few seconds can promote adequate spreading, followed by a longer spin (e.g., 60 s) at higher speed, while maintaining a relatively low acceleration (e.g., 500 rpm/s).

### 3.2 Charging of the Polymeric Substrate

As presented in Table 1, all polymeric substrates used for electronic implants possess a high surface‐ and volume resistivity, as well as high dielectric strength, properties that make them suitable as an insulating material for flexible electronics. However, the high surface and bulk resistivity of the substrate cause charge buildup and results in deflection of the electron beam and pattern distortion [62, 63]. For insulating substrates, anti‐charging measures are thus sometimes required. The effect of charging may also depend on the thickness of the insulating layer (the polymer film) and the acceleration potential, making it essential to test it for various substrate layer thicknesses and to further adapt in the case of a multilayer system. With a high acceleration potential the electrons penetrate deeper into the substrate, enable mitigation of the electrons as they reach the conducting carrier wafer underneath the stacked polymer layer(s) [19].

Common approaches to mitigate the charging include evaporating a thin (10–20 nm) metal film prior to exposure, followed by selective wet etching before development [64]. Thermal evaporation, not electron beam evaporation, must be used for metal deposition to avoid exposing the resist unintentionally, and the wet etchant's compatibility with the substrate material must be ensured when removing the charge dissipation layer [23, 65]. For easier processing, a conductive polymer can be used which is spin‐coated as a thin layer on top of the resist and readily dissolved in water after exposure before development. Dedicated conductive polymers are commercially available, for example, E‐spacer, which are faster in both deposition and removal, and ensure no exposure to aggressive etchants is needed to strip the layer after patterning. The main drawbacks of these alternatives are that they generally have a short shelf life and pH drift, which can decrease conductivity and cause charge build up, and thus make this alternative less reliable than using a thin metal layer [66]. It should be noted that it might not always be necessary to add such a layer, but the need for an additional charge dissipation layer should be evaluated on a case‐by‐case basis.

Charge buildup and possible heating of the resist/substrate can also be avoided by optimizing the writing order, to reduce beam dwelling in the same area for too long periods of time [21].

### 3.3 Heating of the Polymeric Substrates

During electron‑beam exposure, polymer substrates can be exposed to localized overheating, particularly at high accelerating voltages or within densely patterned regions. Compared to conventional substrates such as silicon, polymers exhibit higher thermal sensitivity and poor heat dissipation due to their low thermal conductivity [12]. Additionally, throughout fabrication, they are repeatedly exposed to elevated temperatures during baking, exposure, and curing.

There are several ways this increased temperature affects the polymer substrate: it might cause expansion in the polymer, distorting the fabricated structures; it can reach the polymer's glass transition temperature, causing cracking, physical deformation such as bubbling, or chemical changes in the polymer [12]. Other effects following inadequate heat dissipation during exposure is changes in the chemical reaction rates of the resist, altering the sensitivity and causing pattern distortion [24, 67]. Among polymeric materials, PI offers exceptional thermal stability, with curing temperatures up to 450°C and decomposition near 500°C (e.g., PI‑2611), making it well suited for micro‑ and nanofabrication [38]. In contrast, PaC is particularly sensitive to heat due to lower thermal conductivity and specific heat capacity compared to the other tabulated polymers. Furthermore, PaC has a glass‑transition temperature of only 60°C–100°C and is susceptible to oxidation and cracking at temperatures as low as 125°C [68]. Typical resist baking conditions (100°C–200°C) therefore pose challenges for its processing. For instance, Scholten and Meng observed damage to the PaC substrate due to thermal effects in both baking and exposure steps, and they mitigated these issues by extending the bake time (20 min) at a reduced temperature (120°C instead of 170°C–180°C) to ensure complete solvent removal without thermal degradation, and the substrate was subsequently exposed at 20 kV to reduce thermal effect due to high acceleration voltage [12].

Furthermore, as presented in Table 1, all the presented polymers also have a non‐negligible thermal expansion, which in theory can be problematic when exposing the substrate to heat. Substrates with a high thermal expansion coefficient (301‐310 ppm/°C), such as PDMS, can crack during soft bake, making conventional microfabrication difficult [69].

## 4 EBL Process Implementation

When developing the process, multiple process parameters are varied to identify combinations that yield the most stable and reproducible results [18]. While some parameters are already selected (e.g., substrate material), others need to be systematically tested in various combinations. The multitude of processes and interacting variables in cleanroom fabrication can introduce variability in processing, reducing the reproducibility of patterned structures. Naturally, the small structural dimensions targeted with e‐beam make these processes more sensitive compared to optical lithography, even though the general principles remain the same. Typical EBL process steps, their key parameters are presented in Table 2, which is focused mainly on the implementation of a lift‐off process. Each step is further described in upcoming sections.

**TABLE 2:** Typical patterning process steps and what are the key parameters of each step and the target outcome.

Process step	Key parameters	Target outcome
Masking	Resist tone (positive/negative) Resist choice Resist layer stack Resist thickness Baking parameters Charge mitigation strategies	Resolution Substrate characteristics Substrate thickness Throughput Process control Process latitude
Development	Developer choice Development parameters Dilution Agitation	Resolution Process control Process latitude Substrate characteristics Solvent characteristics
Exposure	Acceleration potential Dose Beam spot size	Resolution Feature sizes Substrate characteristics Substrate thickness Structure shape and periodicity Alignment requirements Throughput Size bias/proximity effect Process control Process latitude
Metallization	Material Deposition method Resist thickness Material thickness Adhesion layer	Resolution Resistance/impedance
Lift‐off	Lift‐off solvent Lift‐off parameters Agitation/other strategies Time	Resolution Throughput/yield Substrate characteristics Solvent characteristics

### 4.1 Masking and Development

#### 4.1.1 Selection of Resist and Spinning Parameters

Important metrics for resists are sensitivity and contrast. The sensitivity of the resist, defined as the minimum electron dose required for complete solubility change, dictates throughput while the contrast dictates the highest achievable resolution [19]. The sensitivity and contrast depends on several parameters; the resist material, its thickness, the developer, development time and temperature, and exposure parameters [24].

Furthermore, the ideal resist has strong adhesion to the substrate, high sensitivity and contrast, good etch resistance, straightforward processing, high chemical purity and stability, minimal solvent content, low cost, and a high glass‑transition temperature [18, 19]. These performance requirements have guided the development of commercial resists that are specialized for, e.g., high throughput (sensitivity), high resolution or high etch resistance.

Table 3 summarizes common positive and negative EBL resists along with their sensitivity, contrast, etch selectivity, and developers. It should be noted that these parameters are highly process‐dependent and therefore serve only as indicative values. The etch selectivity is here represented for oxygen plasma, since it is used for adhesion promotion before metal deposition on the polymer layer.

**TABLE 3:** Common commercial E‐beam resists and their resolution, sensitivity values, contrast, etch selectivity and common developers.

	Resist	Resolution (nm)*	Sensitivity (µC/cm^2^)	Contrast (γ)	Etch selectivity (O_2_ plasma)	Developer	Lift‐off solvent	Comment
Positive	PMMA 950K	<10 [19, 91]	100 at 20 keV [19, 84]	2.8 at 20 keV [84] 4.6 at 100 keV [85]	104 nm/min (20 sccm, 3 mTorr, 40–50 W) [81]	MIBK:IPA [23, 74], IPA:H2O	NMP/1165, DMSO, Acetone [70, 71]	High resolution patterning, low sensitivity, weak etch resistance.
	ZEP520A	10–23 [19, 85]	30 at 20 keV [19] 60 at 30 keV [88]	4‐5 at 20 keV [86] 8 at 100 keV [85]	64 nm/min (20 sccm, 3 mTorr, 40 W) [82]	ZED‐N50 [75], MIBK:IPA, [76] IPA:H_2_O [76]	NMP, Acetone [71], DMC [72]	High‐resolution patterning, higher sensitivity compared to PMMA
	AR‐P 6200 (CSAR 62)	<10 [26, 77]	55 at 30 keV [77] 140 at 100 keV [26]	14 at 30 keV [77] 5.2 at 100 keV [87]	66 nm/min (20 sccm, 3 mTorr, 40 W) [82]	R 600–546, 600–548, 600–549 [77] ma‐D 525, mr‐Dev 600 [78 ^],^ MF319[79]	1,3‐dioxolane [73]	High‐resolution patterning, higher sensitivity compared to PMMA
Negative	HSQ	<5 [92, 93]	214 keV at 40 keV [78] 2000 at 100 keV [89]	1.82 at 40 keV [78]	Very high [83]	ma‐D 525, mr‐Dev 600 [78]^,^ MF319 [79]	Fluroine‐based etchants [71]	Ultra‐high resolution, excellent etch resistance.
	Ma‐N 2400 series	80 [19]	100–350 [80] at 20 keV 120–600 at 100 keV [80, 90]	2.03 at 40 keV [78]	−	ma‐D 525, ma‐D 331 [80]	NMP/1165, acetone [71]	For mix‐and‐match.

The earliest and still one of the most prevalent resists for electron‐beam lithography (EBL) is polymethyl methacrylate (PMMA), valued for its low cost, ease of processing, and well‐understood chain‐scission mechanism enabling sub‐10 nm resolution [19, 94]. Its molecular weight can be tailored to specific process goals where high‐molecular‐weight PMMA (950 K) offers higher resolution but lower sensitivity, whereas low‐molecular‐weight PMMA (495 K) exhibits greater solubility. The primary limitations of PMMA are its low sensitivity and poor etch resistance, which can constrain plasma processing before metal deposition. Bilayer lift‐off schemes commonly combine PMMA with PMGI (LOR), P(MMA‐MAA), or lower‐molecular‐weight PMMA to achieve the undercut profile required for efficient lift‐off [12, 94, 95, 96]. To improve throughput and reduce exposure times, one can utilize more sensitive resists, such as the ZEP 520 or AR‐P 6200 resist series, which also provides improved etch resistance compared to PMMA [26].

As a negative resist, HSQ provides very high resolution in combination with high etch selectivity, but with a downside of having short shelf life and fast degradation after spin‐coating [19, 83, 97]. Alternatively, *mix‐and‐match* lithography integrating EBL and photolithography can be employed [98]. Hybrid negative resists, such as the ma‐N 2400, contain both electron‐ and photon‐sensitive components, enabling pattern transfer for both fine and large‐scale features within the same material [99].

Spinning parameters govern the resist thickness, which strongly influences the resolution and aspect ratio of the patterned features, and the baking conditions ensure adhesion, yield, and reproducibility during development [19, 23]. The recommended spinning and baking condition is usually specified for the resist.

The EBL process that is described and presented as examples in this article uses PMMA 950K as the top layer resist (4000 rpm, 60 s, baked at 160°C for 5 min), copolymer EL 6 as the bottom layer resist (4000 rpm, 60 s, baked at 160°C for 5 min) to facilitate the undercut necessary for a clean lift‐off.

#### 4.1.2 Charge Mitigation Layer

**FIGURE 7:**
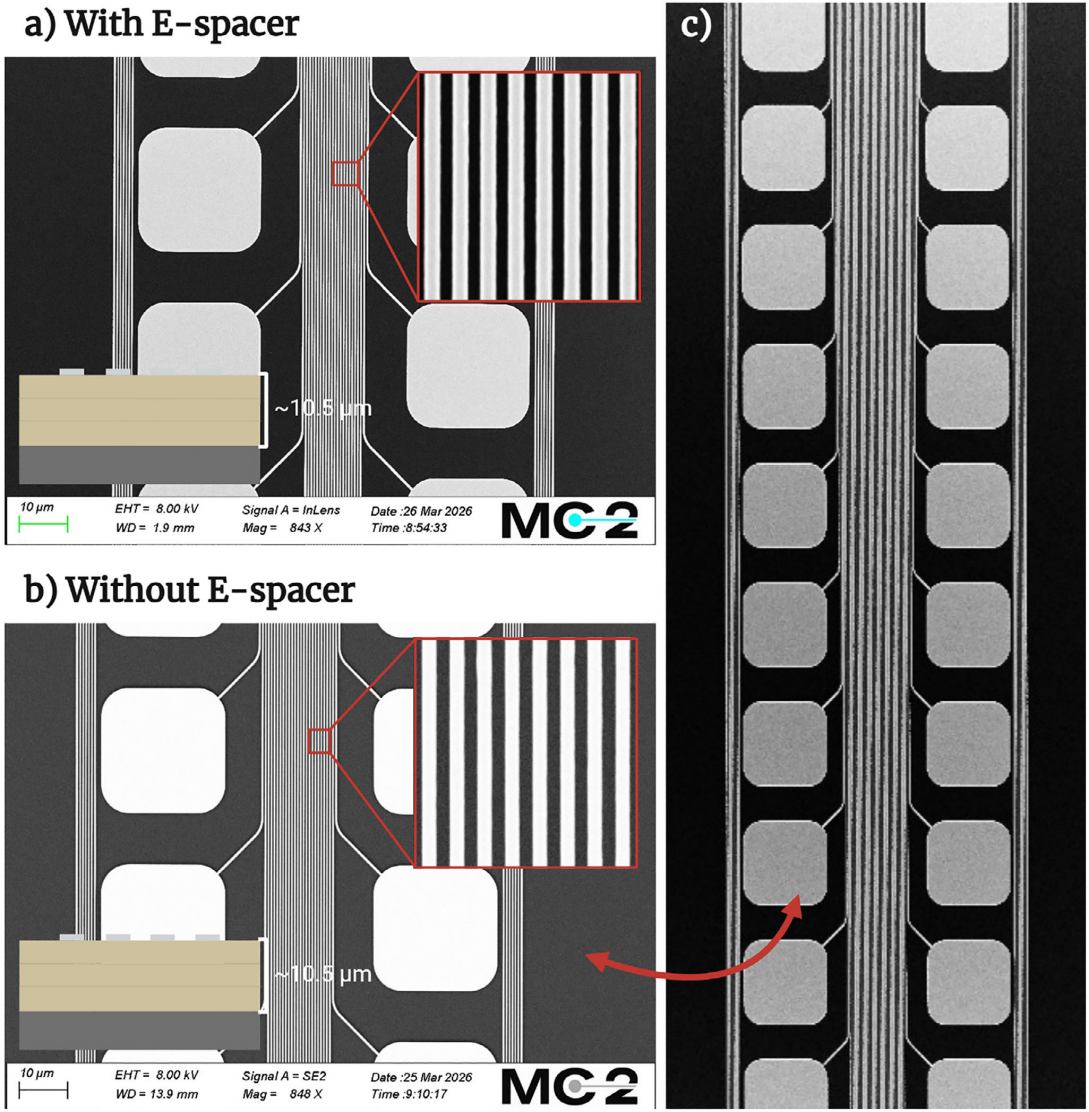
SEM micrographs of samples with a PI thickness of approximately 10.5 µm, with and without E‐spacer as a charge mitigation layer. The line width for the sample with charge mitigation is approximately 306 ± 11 nm and the sample without charge mitigation layer 266,6 ± 7,27. Intended line width is 300 nm and pitch is 600 nm.

Depending on, for instance, the substrate, substrate thickness, and acceleration voltage, a charge mitigation layer might be required. However, here we show that EBL was implemented (at 100 kV) for PI and showed no need for charge mitigation layer (e.g. no E‐spacer layer, as otherwise would have been recommended). Even when sub‐micron structures were fabricated using EBL (at 100 kV) with a 10.5 µm thick PI substrate, there were no obvious signs of charging or distortion of the structures. Figure 7 shows structures fabricated with and without a charge mitigation layer, showing only a difference in the linewidth, 306 ± 11 nm when using E‐spacer and 267 ± 7.3 without E‐spacer. Hence, given the added process steps, and that it might not be necessary when exposing the polymer substrate, charge dissipation layer can be applied selectively.

#### 4.1.3 Development

The development parameters are essential when fabricating high‐resolution structures. Diluting the developer reduces undercutting and prevents unintended dissolution of unexposed areas (also referred to as dark erosion), which is critical when fabricating high‐resolution structures [100]. Moreover, development time influences feature size, line‐edge roughness (described in Section 2.2 EBL challenges), and pattern fidelity. Agitation, such as stirring or ultrasonication, can help improve resolution by releasing trapped molecules for dissolution [19]. When using ultrasonication, however, it's critical not to use too high a power level, as this can destroy or degrade the exposed pattern. Compatibility between the developer and substrate material must also be ensured [100]. After the development, the wafer instantaneously need to be submerged in a solvent that stops the reaction, as if the development continue it can cause overdevelopment that reduces the pattern fidelity. The stop solvent could be, for instance, H_2_O or IPA.

Developers for common EBL resists are shown in Table 3. The developer used for the work shown as examples in this paper is IPA:H_2_O with a ratio of 80:20. The wafer is developed for 2 min, and the wafer is subsequently submerged in a beaker of IPA to stop the reaction and finally dried using blow drying.

### 4.2 Exposure

The exposure parameters define resolution and pattern fidelity and influence thermal effects and electrical charging during exposure. Exposure parameters that need to be selected and optimized are acceleration potential, dose and beam spot size.

In the examples included here, Raith EBPG 5200 is used.

#### 4.2.1 Selection of Acceleration Potential

As described in Section 2.1, achieving a high resolution with EBL, and shown in Figure 2, a high acceleration potential is desired to attain higher resolution. Higher acceleration potential can also help reduce charging, as the electrons penetrate deeper into the polymeric substrate and thus might reach the conducting carrier wafer and be dissipated away.

In the examples included here, an acceleration potential of 100 kV is used. For PI, as shown in this article, this acceleration potential did not cause excessive charging nor damage due to heating. For heat‐sensitive substrates such as PaC, however, a reduced acceleration potential (10‐20 kV) might be necessary.

#### 4.2.2 Selection of Dose Parameters

A dose test is performed to determine the optimal dose for the structures. During the dose test, the electron tbeam exposure is systematically varied across a predefined range to identify doses that yield the desired structures. Test patterns must incorporate critical features representative of the final design, such as those with specific dimensions, geometries, or periodicities, as well as larger elements like contact pads, to verify process compatibility across varying size scales and pattern densities. It is important to keep in mind that a higher dose can also contribute to increased heating or charging of the substrate.

#### 4.2.3 Optimizing Beam Spot Size

**FIGURE 8:**
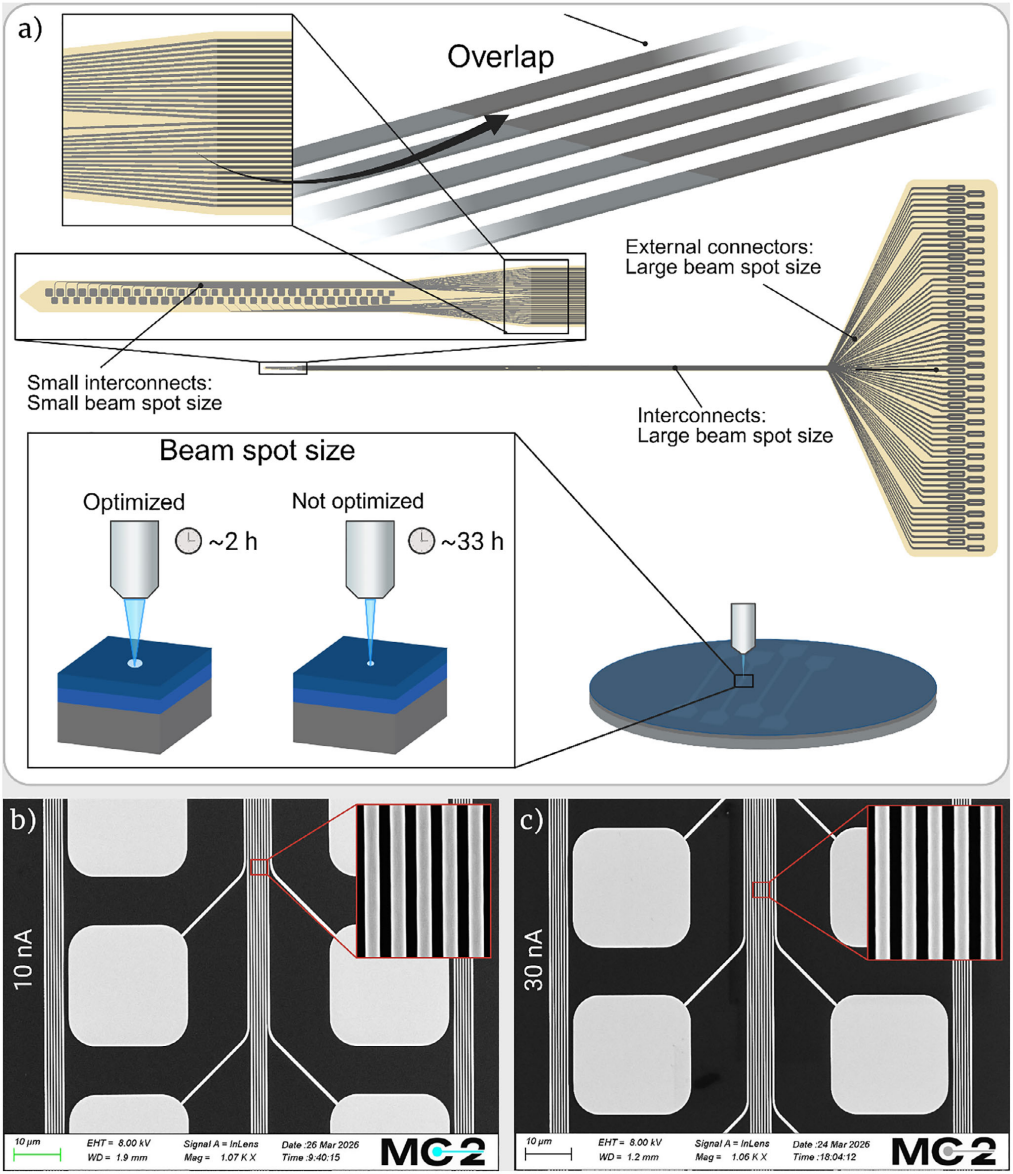
(a) Schematic showing optimization of beam spot size, dividing the design into layers, where the small features use a smaller beam spot size while the large features use a large beam spot size. It shows the overlap where the two exposed regions meet with a slight overlap misalignment due to drifting during exposure. By using this method, the beam spot size can be optimized, and the exposure time can be reduced from ∼33 to ∼2 h. (b) Fabricated structures with a beam current of 10 nA. The measured line width is 299 ± 7,1 nm. The intended line width is 300 nm and pitch is 600 nm. (c) Fabricated structures with a beam current of 30 nA. The measured line width is 307 ± 5,6 nm. The intended line width is 300 nm and pitch is 600 nm.

The beam spot size can be optimized based on the current and dose. and it is critical for increasing throughput. Optimizing beam spot size minimizes drifting, improves alignment accuracy, and reduces overlay errors during the same exposure and between successive layers (Figure 4a–c). The beam spot size can be adjusted according to the feature size and shape. When exposing complex designs featuring interconnects that are only a few hundred nanometers wide to large pads measuring several hundred micrometers, the features are divided already into the design phase and exposed with different beam spot sizes, optimized for beam current and dose (illustrated in Figure 8a). Structures fabricated with different beam spot sizes (10 and 30 nA) are shown in Figure 8b,c, showing no significant difference (299 ± 7,1 nm vs. 307 ± 5,6 nm, respectively) between the line width of the structures. 30 nA was the highest possible beam current for the corresponding dose. This demonstrates that a larger beam spot size can substantially reduce exposure time without compromising the critical feature size. In the example presented in Section 2, E‐beam lithography, the exposure time could be reduced from 3.5 h to 70 min by adjusting only the beam spot size (10 vs. 30 nA). This is advantageous both for production throughput and for minimizing beam drift, thereby improving structural fidelity. For larger structures (larger interconnects and pads) the beam spot size of over 100 nm can be selected, corresponding to a beam current of >200 nA.

In the examples shown in this article, a dose of 300 µC/cm^2^ and a beam current of 10 nA is used for the smaller structures (sub‐µm interconnect) and structures in critical areas (electrodes) whereas a dose of 375 µC/cm^2^ and a beam current of 250 nA is used for the larger structures (µm‐sized interconnects and pads).

### 4.3 Metallization and Lift‐Off

#### 4.3.1 Metallization

Generally, lift‐off is better suited to evaporated material layers due to their higher directionality [101]. The resist layer needs to be sufficiently thick to ensure no bridging between the metal layers and prevent fencing. It is common practice to perform a plasma treatment step prior to material deposition to improve adhesion of metal to the polymer film, which can partially remove the resist, especially for less etch‐resistant resists such as PMMA [3, 11]. Therefore, before the plasma treatment, the resist must be sufficiently thick to enable a clean lift‐off.

Furthermore, the quality of the lift‐off process strongly depends on the properties of the deposited material. To ensure robust adhesion to the underlying polymer substrate, another important aspect of a clean lift‐off and a functioning device, it is advisable to incorporate an appropriate adhesion layer beneath the metallization [102].

The structures shown as examples in this article are metallized with 10 nm Cr (adhesion layer) and 90 nm Pt.

#### 4.3.2 Lift‐Off

Solvent choice is equally essential during lift‐off. N‐Methyl‐2‐pyrrolidone (NMP) or NMP‐based solvents effectively dissolve most resists [103]. Less toxic alternatives are dimethyl sulfoxide (DMSO) can dissolve most resins and are thus more frequently used to replace NMP and NMP‐based solvents [104]. Weaker solvents such as acetone, isopropanol, and methanol are also used for lift‐off but cannot dissolve all resists, for example, PMGI‐based resists. The recommended lift‐off solvents for common resists are summarized in Table 3. It is important to ensure solvent compatibility with the polymer, as some solvents tend to swell or attack polymers [105, 106, 107]

Lift‐off can be passive (soaking for hours) or active (heated solvent with agitation or ultrasonication). The time for lift‐off generally depends on how well the resist is dissolved in the solvent. For strong enough solvents, active lift‐off might not be required, and for delicate sub‐micron features, aggressive methods risk damaging the metallization that is already fragile due to less area for it to adhere to. Gentle solvent squirting via pipette often suffices for complete excess metal removal around fine, repeating structures.

DMSO is used for the structures used as examples in this article for lift‐off, where the wafer is soaked for a couple of hours. The lift‐off is further performed without agitation to minimize the risk of damaging the structures.

### 4.4 Evaluation of the Exposure Parameters and the Fabricated Features

#### 4.4.1 High‐Resolution Imaging Methods

Techniques used to resolve sub‐micron features include atomic force microscopy (AFM), scanning electron microscopy (SEM), and focused ion beam‐SEM (FIB‐SEM). AFM is well suited for measuring three‐dimensional topography because of its sub‐nanometer vertical resolution and its ability to map local physical properties, but it is less suitable for line‐width measurements because lateral dimensions are distorted by tip‐sample convolution, making these measurements less accurate [108]. SEM is often the preferred technique for rapid line‐width analysis, offering nanometer‐scale resolution, non‐destructive imaging, and high throughput [109]. However, SEM can be affected by charging on insulating substrates, which may necessitate the use of a charge mitigation layer, such as a sputtered metal coating or a conductive polymer coating. FIB‐SEM uses a focused ion beam to cross‐section the sample before SEM imaging [110], making it especially useful for examining buried features, such as a metal line between two PI layers. Its limitations are that it is destructive, slower than SEM alone due to the cross‐sectioning before analysis, and susceptible to charging artifacts.

##### 4.4.1.1 SEM Analysis

Following the dose test, exposure, and development, structures are typically metallized to enable compatibility with the entire process, from exposure to metallization and lastly lift‐off. This is to facilitate SEM inspection, which is often difficult on uncoated resist due to charging artifacts [18].

**FIGURE 9:**
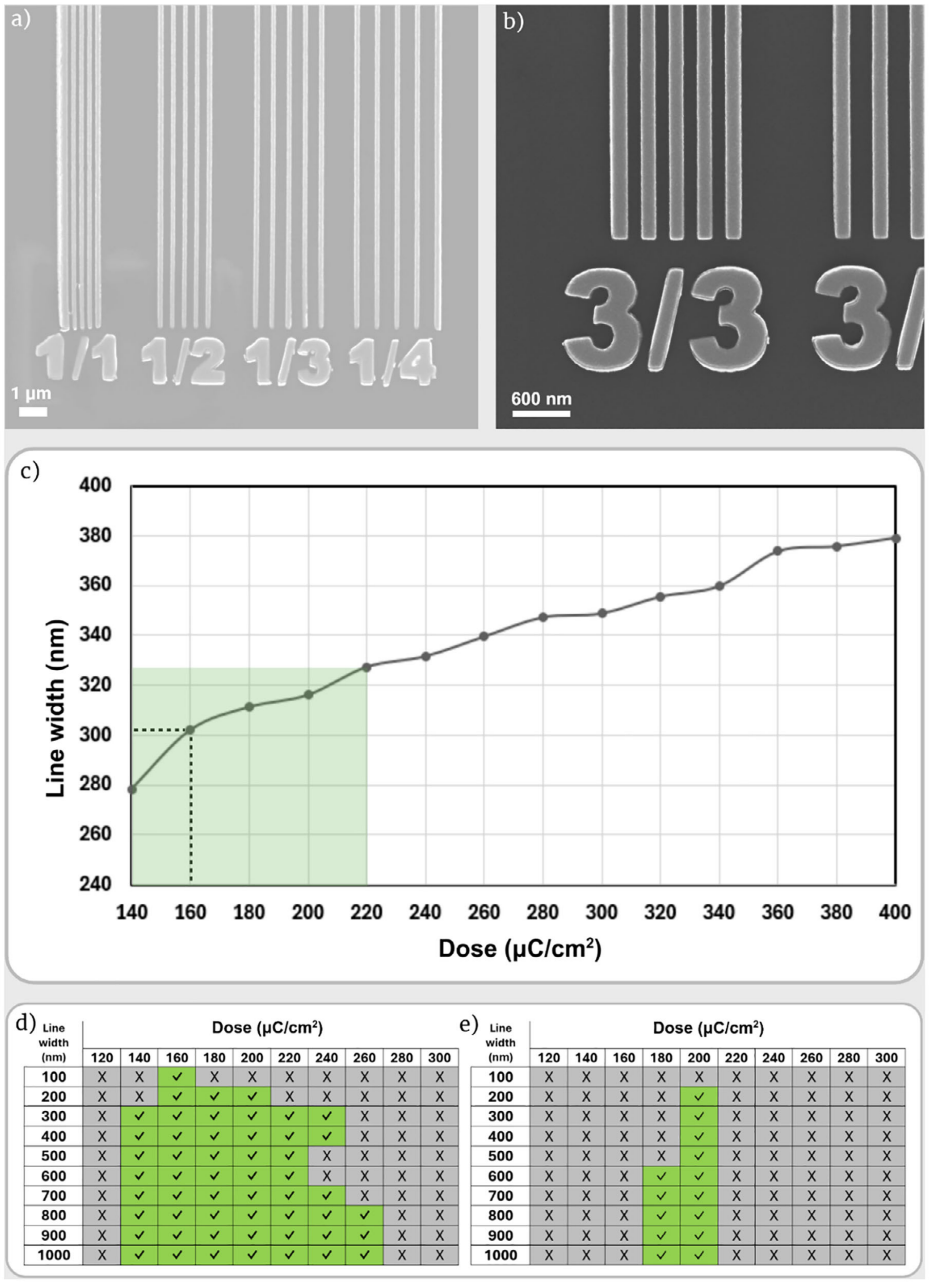
(a) Lines down to 100 nm width/200 nm ‐pitch (1/1 in the image). (b) The test structure used measure the line width as a function of dose. The designed line width is 300 nm, and pitch is 600 nm. The measured line width in the structure is 295 nm. (c) The measured line width of the test structures as a function of dose. The doses that correspond to the intended line width (300 nm) ± 10% is shown in green. Only one dose corresponds to the designed line width of 300 nm. (d) A table showing the process window as a function of dose. The green marked in the table represents doses where the desired line width is attained within ± 10% of the line width. (e) The process window when changing one parameter; here a thicker resist underlayer is used compared to in (d).

An example of a structure for SEM inspection is shown in Figure 9a,b, showing test structures with a line width and pitch down to 100/200 nm (Figure 9a) (measured line width of the 1/2 lines is 97 nm) and larger lines, approximately 300 nm (measured line width 295 nm) (Figure 9b). The corresponding line width to dose for the structure is shown in Figure 9c, and the structures used to determine this are shown in Figure 9b. The SEM images were acquired using a ZEISS Supra 60 VP (ZEISS, Germany), and line width is measured using the built‐in software to the SEM tool (SmartSEM). The intended line width is 300 nm, but the resulting line width (assessed manually with SEM) varies between 280–380 nm. The doses that correspond to a line width within 10% of the intended line width are shown in the green area of the graph. Stable feature dimensions across consecutive doses indicate a robust process, reflecting the nonlinear dose–feature size relationship modulated by development time; steeper slopes in dose–feature size plots denote higher process sensitivity and thus narrower margins, while shallower gradients signal greater tolerance. Thus, usually the dose is selected that yields the highest process stability, and a size bias is applied to accomplish the intended line width.

The process window involves a wide range of parameters that define the whole process, but in practice, it is usually reduced to involve only one or a few parameters, for example, dose. The process window can be directly extracted from SEM analysis of the dose test structure (shown in Figure 9a) as the parameter window showing where line width is preserved over a range of doses. Figure 9b,d shows process windows that define the doses that accomplish certain feature sizes of interconnects (+−10% of the line width, assessed manually with SEM). The primary difference between the process windows shown in Figure 9c,d is the thickness of the resist underlayer. The difference between the process represented in the process windows in Figure 9c,d is that in Figure 9d a thicker resist underlayer is used, where copolymer EL10 is utilized to accomplish an underlayer that is approximately 375 nm compared to approximately 135 nm with copolymer EL6, else all other process parameters remain unchanged. A thicker underlayer can improve lift‐off performance and enable deposition of thicker metallization layers, which is critical for maintaining adequate conductivity, particularly in miniaturized features. However, the results also illustrate how sensitive the process window is to this single parameter, as increasing the underlayer thickness leads to a noticeably narrower process window.

#### 4.4.2 Electrical Measurements

**FIGURE 10:**
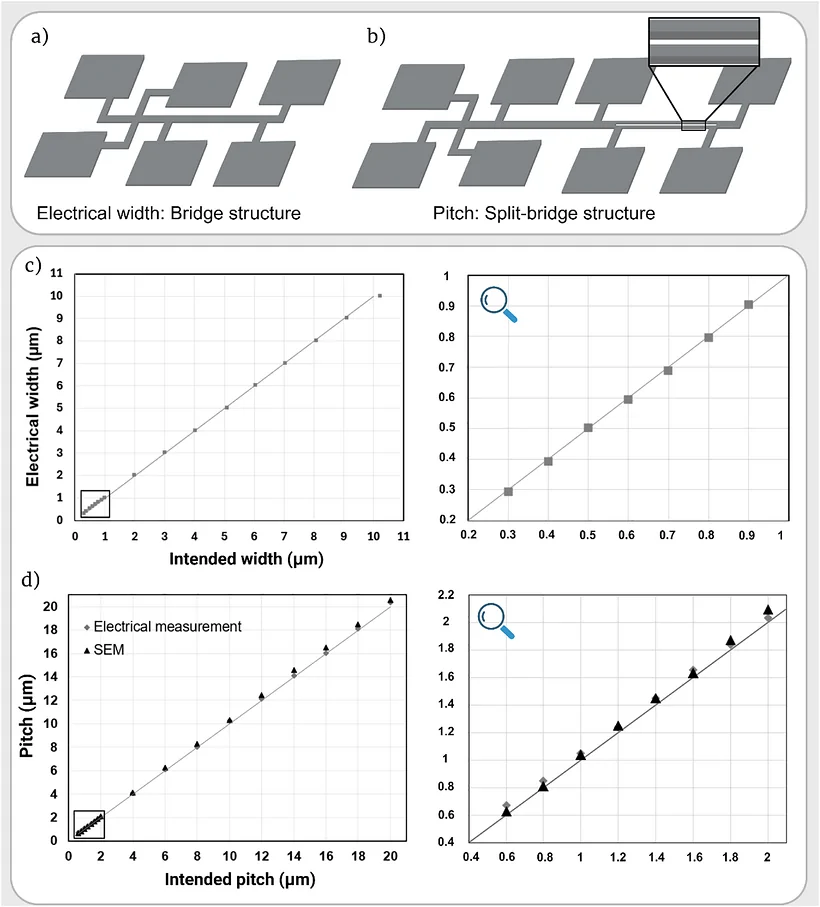
(a) The bridge structure which is used to measure and calculate the electrical width of single lines. (b) The split bridge structures for measuring and calculating the electrical pitch of double lines. (c) The measured and calculated electrical width of the bridge structure, with line widths varying from 0.3 to 10 µm. The measured line width corresponds well with the intended line width. (d) The measured and calculated electrical pitch of the split bridge structure, with line width varying from 0.3 to 10 µm, and pitch varying from 0.6 to 20 µm. The electrical measurements are also supported by SEM measurements, showing correspondence between the fabricated structures, which also corresponds with the digital pitch.

Electrical linewidth determination offers a complementary, quantitative alternative to deducing the line with and pitch via four‐point resistance measurements on dedicated bridge and split‐bridge test structures [111]. Schematic of the bridge and split‐bridge structures are shown in Figure 10a,b. With only a series of four‐point probe measurements and calculations one can deduce the electrical width and pitch of the single and double lines. The structures, their dimensions, measurement and calculation procedure can be found in Andreas Tsiamis doctoral thesis [111]. Example of the measured and calculated electrical width and pitch of fabricated bridge‐ and split bridge structures are shown in Figure 10c,d. Structures with a line width of 300 nm of the bridge structure and 600 nm pitch of the split‐bridge structure were fabricated and measured using a four point probe station (Keithley 4200‐SCS, Tektronix). Figure 10c presents the measured electrical width of the bridge structures as a function of the designed line width, ranging from 300 nm to 10 µm, fabricated using the same process. The results exhibit a linear relationship that closely matches the intended dimensions. Similarly to the electrical width, the measured pitch of the bridge structure followed the same linear relationship across the line width. Notably, achieving the target line width requires the application of a size bias, meaning that the structures were intentionally designed with smaller dimensions to compensate for process‐induced broadening, resulting in final widths that align with the intended values.

The electrical measurement of the pitch of this structure was also backed up by SEM measurement of the line width, that shows a high correspondence to the intended line width as well as the electrical measurement. The pitch was manually measured by using a built‐in software SmartSEM to the ZEISS Supra 60 VP SEM tool. These measurements show that using electrical measurements for validation of line width and pitch is valid.

## 5 Discussion and Conclusion

While EBL is an established nanofabrication technique, relatively limited data are available on its use with polymeric substrates. Nevertheless, EBL has the potential to advance neurotechnology, provided that sufficient effort is devoted to implementing, characterizing, and optimizing the nanofabrication processes required for these devices. High channel density is advantageous in BCI/BMI systems because it increases spatial sampling of neural activity, improves decoding of complex signals, and enhances information transfer and robustness. With this review, we aim to contribute to the understanding of thin‐film fabrication and EBL, and to support their implementation for the fabrication of nanoscale electrical features.

Patterning polymeric substrates by EBL is a multifactorial optimization problem, as discussed in this article. Interactions to consider are substrate non‐planarity, heat generation during exposure, and charging of the insulating polymer layer. Despite these challenges, EBL can reproducibly fabricate structures down to 50/200 nm width/pitch [13] and, in our own experience we have achieved features down to 100/200 nm width/pitch on PI using the methods outlined here. With an acceleration voltage of 100 kV there was even no need for a charge‐mitigation layer, pointing to the importance of optimizing the process for the specific polymer, resist, design and EBL system.

The inherently slow writing speed of EBL is widely regarded as its primary limitation, particularly for large‐area patterns and full‐wafer exposures. Consequently, as described in this review, selecting a resist that enables rapid patterning, together with optimization of exposure parameters, especially the beam spot size, is critical, as it reduces overall exposure time while improving feature fidelity by minimizing beam drift.

Even if this review emphasizes the complexity, and trade‐offs to be made in the process design, we would like to lift that fabrication of nanoscale features on polymeric substrates may in some cases be less challenging than initially assumed. Investing in systematic process optimization early on and working with carefully selected teststructures may save on both time, cost and complexity downstream, and open the possibility for wider use of EBL in the fabrication of miniaturized multichannel electronic implants.

EBL is shown to be a powerful technique for miniaturizing device features and increasing channel density, and its feasibility for fabricating neural implants has already been demonstrated. However, careful evaluation of the implant hardware remains essential to ensure reliable conductivity of the miniaturized interconnects, strong metallization adhesion, and adequate electrical performance, including low crosstalk. In addition, increasing the number of channels introduces further challenges related to connectors, data transmission, and power delivery, all of which must also be miniaturized while maintaining long‐term reliability. With sustained progress in addressing these issues, BCI/BMI systems could eventually have a substantial clinical impact.

## Author Contributions

**Hanna Karlsson‐Fernberg**: investigation, conceptualization, writing – original draft, methodology, writing – review and editing, formal analysis, data curation. **Maria Asplund**: conceptualization, investigation, funding acquisition, methodology, validation, visualization, writing – review and editing, project administration, supervision, resources.

## Funding

This work was funded by the European Union Horizon‐EIC‐2022‐Pathfinder Open program under grant agreement No. 101099366 (BioFINE) and furthermore, the Chalmers Gender Initiative for Excellence (GENIE).

## Conflicts of Interest

The authors declare no conflicts of interest.

## Data Availability

The data that support the findings of this study are available from the corresponding author upon reasonable request.
